# SYSTEMATIC REVIEW OF PHYSICAL ACTIVITY AND SEDENTARY BEHAVIOR INDICATORS IN SOUTH-AMERICAN PRESCHOOL CHILDREN

**DOI:** 10.1590/1984-0462/2020/38/2018112

**Published:** 2019-11-25

**Authors:** Paulo Henrique Guerra, Valter Cordeiro Barbosa, Angélica Almeida, Letícia de Souza Silva, Marcelo Tedesco Vidal Pinto, Renan Martinelli Leonel, Evelyn Helena Corgosinho Ribeiro, Alex Antonio Florindo

**Affiliations:** a Universidade Federal da Fronteira Sul, Chapecó, SC, Brazil.; b Instituto Federal do Ceará, Boa Viagem, CE, Brazil.; c Universidade de São Paulo, São Paulo, SP, Brazil.

**Keywords:** Physical activity, Sedentary lifestyle, Indicators (Statistics), Child, Review, Atividade física, Estilo de vida sedentário, Indicadores, Criança, Revisão

## Abstract

**Objective:**

To summarize indicators and describe in detail the methods used to physical activity and sedentary behavior measurement in South American preschool children.

**Data source:**

In 2017, we searched for articles on researches carried out in South American countries, which presented physical activity and/or sedentary behavior indicators in children aged two to six years. These searches were conducted in Spanish, English, and Portuguese in four electronic databases (LILACS, PubMed, SciELO, and Web of Science), Google Scholar, and in reference lists.

**Data summary:**

Out of 416 articles initially assessed, 13 composed the descriptive summary. Samples from Argentina, Brazil, and Chile were included. Three articles provided accelerometer-based estimates of moderate physical activity: 32, 70.1, and 71.3 minutes per day. The mean total sedentary behavior was 468.3 and 562.9 minutes per day and, considering the cut-off point of 2 hours per day of screen time, we found the following prevalence rates: 39.4, 40.3, and 100%. The studies adopted a wide number of instruments and strategies to evaluate those behaviors.

**Conclusions:**

Although the summary has pointed to high exposure to sedentary behavior in preschool children, with particular focus on screen time, it is essential to broaden the discussion and approximate the methods used to assess physical activity and sedentary behavior, making the evidence more comparable and strong, so as to elaborate preventive strategies and actions.

## RESUMO

**Objetivo:**

Sumarizar indicadores e descrever detalhadamente os métodos utilizados na avaliação da atividade física e do comportamento sedentário em pré-escolares sul-americanos.

**Fontes de dados:**

Em 2017, buscas foram realizadas, nos idiomas espanhol, inglês e português, em quatro bases de dados eletrônicas (LILACS, PubMed, SciELO e Web of Science), no Google Acadêmico e em listas de referências. Foram procurados artigos sobre pesquisas realizadas em países da América do Sul, que apresentaram indicadores de atividade física e/ou de comportamento sedentário em crianças entre dois e seis anos de idade.

**Síntese dos dados:**

De 416 artigos inicialmente avaliados, 13 compuseram a síntese descritiva. Foram envolvidas amostras da Argentina, Brasil e Chile. Três artigos ofereceram estimativas médias de atividade física moderada, por meio de acelerômetros: 32; 70,1 e 71,3 minutos por dia. Sobre o comportamento sedentário total, foram observadas as médias de 468,3 e 562,9 minutos por dia e, considerando-se o ponto de corte de 2 horas por dia em comportamentos de tela, as seguintes prevalências foram encontradas: 39,4; 40,3 e 100%. Observou-se grande variação no número de instrumentos e estratégias utilizadas para a avaliação dos comportamentos.

**Conclusões:**

Mesmo que a síntese tenha apontado para elevadas exposições ao comportamento sedentário em pré-escolares, com particular enfoque nas exposições ao tempo de tela, é importante um maior debate e aproximação dos métodos empregados na avaliação da atividade física e do comportamento sedentário, o que tornaria suas evidências mais comparáveis e fortalecidas para a formulação de estratégias e ações preventivas.

## INTRODUCTION

The preschool period is marked by the potential growth and development of a person. The introduction of healthy behaviors in this life stage is critical, as it increases the chance of these behaviors persisting throughout life.^1^ ^,^ ^2^

Nonetheless, recent evidence points to low levels of moderate and vigorous physical activity^3^ and excessive exposure to screen time, considering recommendations that suggest the cut-off point of less than two hours of daily recreational (or non-educational) screen time.^4^ ^,^ ^5^ We emphasize that recreational screen time is one of the most common indicators of sedentary behavior in studies conducted with children and adolescents.^6^ ^,^ ^7^

Besides the recognized physical, psychological, social, and cognitive benefits associated with the practice of regular physical activity in childhood,^8^ ^,^ ^9^ it is noteworthy that the topic of sedentary behavior is becoming more prominent, particularly due to the awareness of its risk associations with body composition, psychosocial health, and cognitive development^10^ ^,^ ^11^ in preschool children, with the latter two variables presenting potential dose-response relationships.^10^

Considering that most researches that substantiate the knowledge available were conducted in North American and European countries and Australia and that socioeconomic status is an important determinant of development in preschool age,^12^ the gathering and discussion of indicators of physical activity and sedentary behavior from other locations worldwide become valuable. In this regard, South America stands out as a place of interest, both for the increased prevalence of inactive adolescents and their high exposure to recreational screen time in various parts of the continent^13^ ^,^ ^14^ and for being an important research development center on the topic.^15^

Thus, the present study aimed to identify and compare indicators of physical activity and sedentary behavior in South American preschool children, as well as describe in detail the methods adopted to measure these behaviors.

## METHOD

This study is a systematic review, designed, conducted, and reported based on items from the Preferred Reporting Items for Systematic Reviews and Meta-Analyses checklist (PRISMA).^16^ This systematic review was not registered.

As inclusion criteria, we searched for original articles, with observational design, conducted in South American countries, without requiring a representative sample, and presenting indicators of physical activity and/or sedentary behavior in preschool children, classified, for this study, as those aged two to six years. On the other hand, we excluded articles involving samples of children with disabilities and/or clinical conditions in common (e.g., diabetes), except for those comprising overweight and/or obese children.

In 2017, we conducted systematic searches in Spanish, English, and Portuguese in four electronic databases - Latin American and Caribbean Health Sciences Literature (LILACS), PubMed, Scientific Electronic Library Online (SciELO), and Web of Science -, following the strategies designed by PubMed (searching for terms in the body of the text), both for physical activity: Argentina or Bolivia or Brazil or Chile or Colombia or Ecuador or Guyana or Paraguay or Suriname or Uruguay or Venezuela and “physical activity” and toddler or infant or preschool; and sedentary behavior: Argentina or Bolivia or Brazil or Chile or Colombia or Ecuador or Guyana or Paraguay or Suriname or Uruguay or Venezuela and “sedentary behavior” or “sitting time” or “screen time” or “television time” or “computer time” or “video game time” and toddler or infant or preschool. The corresponding author can provide a complete description of the searches. As an additional strategy, we searched Google Scholar and the reference lists of the articles submitted to data extraction.

Next, four previously trained researchers independently evaluated the headings and abstracts, full-texts, and data extracted, with the aid of a senior researcher, to solve potential doubts and establish consensus throughout the process. All references retrieved from the databases were evaluated (by their headings, abstracts, and full texts) simultaneously. The assessment of headings and abstracts was based on four inclusion criteria (objective of the research, study location, age group, and sample characteristics) and conducted in a sensitive manner to avoid potential losses, keeping in the evaluation process not only potentially relevant articles but all studies that could be eligible for subsequent checking of their full texts.

We extracted the original data and included them in an electronic spreadsheet, organized into three domains:

- Descriptive information (research location, year of collection, sampling procedures, sample size, percentage of girls in the sample, age group, mean age, and objective of the study).
- Methodological information (instruments used to assess physical activity and sedentary behavior, domains and types evaluated, and criteria and cut-off points adopted to classify the level of physical activity and sedentary behavior).
- Measurements and indicators of physical activity and/or sedentary behavior (prevalence, time per day) and additional results (data from subgroup analyses and associations among variables). At the end of data extraction, we elaborated a descriptive summary, separating the results by physical activity and sedentary behavior topics.

The risk of bias of the original articles was assessed using an adapted version of the instrument Quality Assessment Tool for Quantitative Studies of the Effective Public Health Practice Project (EPHPP),^17^ which covers the domains:

- Selection bias (sample information, whether heterogeneous or specific for a clinical condition).
- Study design (sample representativeness and sampling methods used).
- Instruments to assess physical activity and/or sedentary behavior (prior validation of the instrument and information enabling the replication of the measurement).
- Losses and withdrawals (information about losses and withdrawals, as well as the percentage of children who had their data analyzed).
- Analysis (suitability of statistical methods used in the research).

## RESULTS

**Figure 1:**
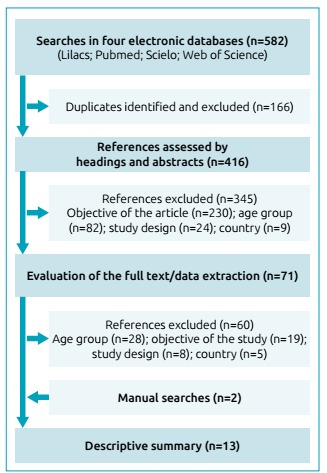
Flowchart of the systematic review.

We retrieved a total of 582 articles in electronic searches (Figure 1). After identifying and excluding the duplicates (n=166), 416 potential articles had their headings and abstracts evaluated. At the end of this stage, we excluded 345 articles, due mainly to discrepancies regarding the objectives (n=230) and age group (n=82). Among the 71 remaining articles that had their full texts assessed, 60 were excluded, mainly for inconsistencies related to age group (n=28) and objectives (n=19). Thus, 11 articles were submitted to data extraction. After the inclusion of two articles retrieved in manual searches, the descriptive summary consisted of a total of 13 original articles.^18^ ^,^ ^19^ ^,^ ^20^ ^,^ ^21^ ^,^ ^22^ ^,^ ^23^ ^,^ ^24^ ^,^ ^25^ ^,^ ^26^ ^,^ ^27^ ^,^ ^28^ ^,^ ^29^ ^,^ ^30^

The study design determined that all included articles had a cross-sectional nature. In all, the summary gathered data from 11 different samples: five from Chile, five from Brazil, and one from Argentina. Studies by Lima et al.^24^ and Melo et al.^26^ used the same sample from Recife (Brazil), assessed in 2010, similarly to Vásquez and Salazar^28^ and Vásquez et al.,^29^ who analyzed the same sample from Santiago (Chile). Five samples adopted randomized techniques for their composition, and the study by Roda et al.^27^ involved children from all child development centers in the city of Merlo (Argentina). The sample size ranged from 24^28^ ^,^ ^29^ to 1,020 children^26^ (Table 1).

**Table 1:** Descriptive characteristics of the articles included (n=13).

References	Local (year of collection)	Sampling technique	Sample size (% of girls)	Age group (years)
Aguilar-Farías et al.^18^	Temuco, Chile (nd)	Randomized	25 (52)	4-5
Barbosa et al.^19^	Londrina, Brazil (2015)	Randomized	370 (50)	4-6
Barro et al.^30^	Olinda, Brazil (2004)	Randomized	265 (54)	4-6
Bielemann et al.^20^	Pelotas, Brazil (2015)	Randomized	59 (nd)	4-5
Cano Cappelacci et al.^21^	Metropolitan Area of Chile (nd)	nd	29 (57)	5
Cremm et al.^22^	Santos, Brazil (nd)	Randomized	302 (48)	<6
Godard et al.^23^	Santiago, Chile (2006)	Convenience	109 (nd)	4-10
Lima et. al.^24^ ^a^	Recife, Brazil (2010)	Randomized	176 (nd)	3-5
Melo et al.^26^ ^a^			1,020 (49)	
Lopez and Llanos; Diaz^25^	Talca, Chile (nd)	Convenience	45 (44)	3-6
Roda et al.^27^	Merlo, Argentina (2014)	All child development centers of the city	183 parents (nd)	1-5
Vásquez and Salazar^28^ ^b^	Santiago, Chile (nd)	Convenience	24 (50)^c^	3-5
Vásquez et al.^29^ ^b^				

^a^Belong to the study ELOS-Pré; ^b^belong to the same study; nd: not described.

Eight of the 13 articles included used accelerometers to measure physical activity and/or sedentary behavior levels (66.7%). However, we noted a high number of devices (n=6) and protocols adopted for their use. Among them, we can mention the significant variation in the number of days used for the measurements (two to seven days), measurements done on weekends (which occurred in three out of five articles with reports available), and different cut-off points to determine sufficient physical activity and sedentary behavior (Table 2).

**Table 2:** Description of the instruments used to measure physical activity and sedentary behavior (n=13).

Assessment instrument and description of its use
Accelerometers
Aguilar-Farías et al.:^18^ ActivPAL micro: four full days (weekdays and weekend). The study evaluated periods of at least 5 or 10 minutes of PA. Estimates of PA and SB for an average day were retrieved from previous studies: mean steps per day=(5 steps per weekday+2 steps per day of the weekend)/7.
Barbosa et al.:^19^ Actigraph GT3X: five consecutive school days. The 75th percentile for PA and SB were adopted as cut-off points. The average number of minutes per day considered valid for the use of accelerometers was at least 360 minutes for children aged 4 to 5 years and 120 minutes for children aged 6 years. Two cut-off points were adopted to classify physical activity and sedentary behavior ^a,b^.
Bielemann et al.:^20^ Actigraph GT1M: full days. The participants were instructed to report on a diary when they did not wear the device for more than an hour. The epoch was adjusted for five seconds, and the accelerometers were delivered in the households on Saturdays and collected on Wednesdays.
Cano Cappelacci et al.:^21^ Actigraph GT3X: the measurements occurred in two weekdays (time: 6 continuous hours).
Godard et al.:^23^ Actiwatch AW64: Sufficient MVPA: number of minutes per day with cpm>900, according to the classification of Puyau et al.^c^ For nighttime PA, all records showing activity (cpm>25) were selected for more than 60 minutes.
Lima et al.:^24^ ^d^ Actigraph GT1M: seven full days (weekdays and weekend). The accelerometer monitoring used epochs of 15 seconds. The cut-off points adopted to set the intensity of the activities performed followed a prior reference^e^. Non-monitoring was determined after 30 consecutive minutes without any count record.
Vásquez and Salazar^28^ and Vásquez.^29^ ^f^ Tritrac R3D: three consecutive days (weekdays and weekend).
Questionnaires
Barros et al.:^30^ adapted version of the questionnaire from the Childhood Obesity Study in Florianópolis.
Cremm et al.:^22^ Children and youth physical activity questionnaire: adapted for the Brazilian population, taking into account the number of daily hours that the child spends on screen behavior, the type of transport the child uses to go to school, and sports practiced.
López et al.:^25^ QDS: parents reported the amount of PA practiced in a week.
Melo et al.^26^ ^d^: QDS: question directed to parents: on a weekday (Monday to Friday), how much time does your child spend playing outdoors, in the garden, the yard, or on the streets near home?
Roda et al.:^27^ QDS: open question directed to parents about the number of hours that their children spend on screen activities. Also, PA was classified as “unstructured”/moderate or “structured”/vigorous.

^a^Sirard et al. J Phys Act Health. 2005; 2:345-357; ^b^Van Cauwenberghe et al. Int J Pediatr Obes. 2011; 6:582-589; ^c^Puyau et al. Obes Res. 2002; 10:150-157; ^d^Belong to the study ELOS-Pré; ^e^Pate et al. Med Sci Sports Exerc 2010; 42(3):508-512; ^f^belong to the same study; PA: physical activity; SB: sedentary behavior; MVPA: moderate and vigorous physical activity; cpm: counts per minute; QDS: questionnaire developed for the study.

The other five articles used questionnaires to evaluate physical activity and/or sedentary behavior, with three of them developing their own instruments,^25^ ^,^ ^26^ ^,^ ^27^ in addition to the use of the Children and Youth Physical Activity questionnaire and the adapted version of the questionnaire from the Childhood Obesity Study in Florianópolis. A common characteristic among the questionnaires is that they were all administered to parents/guardians. On the other hand, we found high variability among the questions, as well as in the approach of physical activity regarding the coverage of weekdays/weekends, places, and intensity of the activities performed (Table 2).

**Figure 2:**
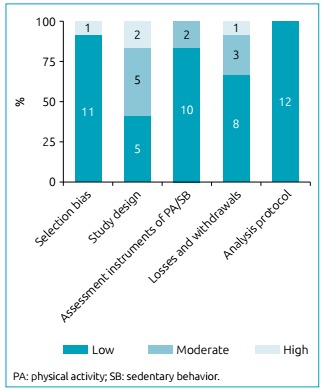
Risk of bias analysis of the articles included.

Since the articles by Vásquez et al.^28^ ^,^ ^29^ used the same sample and techniques to evaluate physical activity, 12 articles were assessed for risk of bias (Figure 2). With respect to selection bias, only the evaluation of the articles by Vásquez resulted in a high risk of bias, as their sample was specifically composed of obese children.^28^ ^,^ ^29^ Given the study design, the most common weaknesses were the lack of information about sample representativeness^18^ ^,^ ^21^ ^,^ ^23^ ^,^ ^24^ ^,^ ^25^ ^,^ ^27^ ^,^ ^28^ ^,^ ^29^ and the use of convenience samples.^23^ ^,^ ^25^ Two articles did not report prior validation of the instrument used to evaluate physical activity^27^ and sedentary behavior.^25^ ^,^ ^27^ Four articles showed a high percentage of losses, considering the difference between the children who had their informed consent form signed and those who were referred to analyses of physical activity and/or sedentary behavior.^20^ ^,^ ^21^ ^,^ ^22^ ^,^ ^28^ ^,^ ^29^ One article did not present information about losses and withdrawals throughout the research.^22^

Three articles estimated the daily volume of moderate physical activity as 32, 70.1, and 71.3 minutes per day.^20^ ^,^ ^24^ ^,^ ^29^ As to the volume of vigorous physical activity, two articles presented the following estimates: 15 and 21.7 minutes per day.^20^ ^,^ ^24^ The mean daily time spent practicing moderate and vigorous physical activity was 92 and 97.1 minutes per day^18^ ^,^ ^23^ (Table 3).

**Table 3:** Indicators of physical activity in South American preschool children.

Mean PA time	
Aguilar-Farías et al.^18^	
Walking	147.2 minutes/day (SD=52)
PA (at least 10 minutes)	97.1 minutes/day (SD=53)
Barbosa et al.^19^	
Light to vigorous PA during school hours	275.2 minutes/day (SD=78)
Bielemann et al.^20^	
Light PA	261.9 minutes/day (SD=37)
Moderate PA	71.3 minutes/day (SD=19)
Vigorous PA	15 minutes/day (SD=8)
Cano Cappelacci et al.^21^	
Eutrophic children	141.6 minutes/day
Overweight children	126.2 minutes/day
Obese children	130.9 minutes/day
Godard et al.^23^	
Moderate and vigorous PA	92 minutes/day (SD=41)
Lima et al.^24^ ^a^	
Moderate PA	70.1 (95%CI 66.2-74.0)^b^
Vigorous PA	24.7 (95%CI 22.3-23.1)^b^
Vásquez and Salazar^28^ and Vásquez^29^ ^c^	
Moderate PA (weekdays)	32 minutes/day
Moderate and vigorous PA (weekends)	22 minutes/day
Prevalence of PA by cut-off point	
Barros et al.^30^ ^q^	
>60 minutes/day of outdoor PA	34.7%
Cano Cappelacci et al.^21^	
>60 minutes/day of moderate and vigorous PA	100%
Cremm et al.^22^ ^q^	18.9%
Godard et al.^23^	
≥60 minutes/day of moderate and vigorous PA	81.8%
Lima et al.^24^ ^a^	
≥60 minutes/day of moderate and vigorous PA	12.7 (95%CI 7.6-19.7)^c^
Melo et al.^26^ ^q^	
>60 minutes/day of moderate and vigorous PA	36.4 (95%CI 33.6-39.2)^d^
Roda et al.^27^ ^q^	
Moderate PA	8%
Vigorous PA	58%

^a^Belong to the study ELOS-Pré; ^b^criteria 3 + days with 10 + h/day (recommended by the authors); ^c^belong to the same study; ^d^measured only on weekdays; PA: physical activity; SD: standard deviation; 95%CI: 95% confidence interval; q: measure originated from a questionnaire.

Considering accelerometer-based measurements, the summary identified the following prevalence rates: 12.7, 81.8, and 100% of preschool children who practice at least 60 minutes per day of moderate and vigorous physical activity^21^ ^,^ ^23^ ^,^ ^24^ (Table 3). In articles that used the questionnaires, we found prevalence rates of 34.7 and 58% of children who practice at least 60 minutes per day of outdoor^30^ and moderate^27^ physical activities, respectively.

Seven articles presented measurements of sedentary behavior based on screen time (n=3), sitting time during school hours (n=2), and the total period of sedentary behavior (n=2) (Table 4). Table 4 shows two accelerometer-based means of daily time of sedentary behavior: 468.3 minutes per day (7.8 hours)^18^ and 562.9 minutes per day (9.4 hours).^20^ Based on questionnaires answered by parents, three articles indicated prevalence of children exposed to at least two hours per day of screen time: 39.4, 40.3, and 100%.^22^ ^,^ ^25^ ^,^ ^27^ Two of these articles also analyzed the prevalence of children exposed to, at least, five hours per day of screen time, with results of 7.2 and 15.5%^25^ ^,^ ^27^ (Table 4).

**Table 4:** Indicators of sedentary behavior in South American preschool children.

Period of sedentary behavior	
Aguilar-Farías et al.^18^	
Total sedentary behavior	468.3 minutes/day (SD=92)
Barbosa et al.^19^	
Sedentary behavior at school	2,234.5 minutes/week (SD=353)
Bielemann et al.^20^	562.9 minutes/day (SD=102)
Prevalence of sedentary behavior by cut-off point	
Barbosa et al.^19^	
School day	89.6-90.9%
Cremm et al.^22^ ^q^	
>2 hours/day of television	39.4%
>1 hour/day of computer and games	27.5%
Lopez et al.^25^ ^q^	
≥2 hours/day of television	100%
≥5 hours/day of television	15.5%
Roda et al.^27^ ^q^	
>2 hours/day of screen time	40.3%
≥5 hours/day of screen time	7.2%

SD: standard deviation; q: measure originated from a questionnaire.

## DISCUSSION

This review aimed at identifying and summarizing indicators of physical activity and sedentary behavior in South American preschool children, as well as the methods used to measure them. Among the evidence found, we underline:

- That most of the articles included presented moderate physical activity levels exceeding 60 minutes per day.
- The high exposure to screen time, considering the cut-off point of two hours per day.
- The high variability of instruments and strategies used to measure physical activity and/or sedentary behavior.

Researches that investigate the lifestyle of preschool children are very important, as their outcomes might not only provide state of the art on the topic but also support the planning and execution of strategies for prevention and/or health promotion, particularly in local communities and social protection networks,^31^ that could control the risk of interference in the development, which, in turn, is also associated with low socioeconomic status.^12^

### Physical activity indicators

Contrary to the present finding, three previous reviews, whose conclusions are mostly based on data from studies conducted in high-income countries, suggest low levels of moderate and vigorous physical activities in preschool children.^32^ ^,^ ^33^ ^,^ ^34^ However, any direct comparison between the findings of this review and the references cited should be weighted, acknowledging the issues related to the representativeness of the samples involved, in addition to the reduced number of South American researches.

Both the tendency of maintaining moderate physical activity between early childhood and pre-adolescence^1^ ^,^ ^32^ and the gradual reduction in physical activity over the years^35^ reinforce the recommendation that strategies and incentives for this practice should be fostered since the first years of life. Thus, further tracking studies should be developed in South America, so this evidence can provide a better understanding of this behavior and deepen the discussion on the specificities of the continent.

In face of the evidence of two previous reviews,^35^ ^,^ ^36^ which suggest gender and the support of parents/guardians as determinants of physical activity, future studies on the theme should intensify their investigations, developing, for instance, stratified analyses that could allow the discussion and comparison of their findings with those based on research conducted in the countries previously mentioned. We also believe that this is an important point, as this expansion in the debate can support the decision making and formulation of public policies that promote physical activity in this population and are suitable to the particularities of a given group, or even a territory.

### Sedentary behavior indicators

In the summary, the lowest prevalence rates of preschool children exposed to at least 2 hours of screen time per day were 39^22^ and 40%.^27^ Given the increase in exposure to sedentary behavior in the transition between childhood and adolescence^37^ and the different negative health indicators associated with high exposure,^11^ previous interventions are important and have significant effects on its control, especially when they involve strategies that monitor screen time, counseling, and participation of parents/guardians.^38^

Considering the socioeconomic characteristics of the South American continent, it is also essential to understand that various socioeconomic aspects are associated with the persistence of sedentary behavior in childhood, and take these aspects into account while formulating preventive strategies.^39^ Therefore, further research on the topic in different scenarios is necessary, so that the evidence can strengthen action plans and strategies on a larger scale.

One of the articles included in this summary presents data collected in the school environment.^19^ Besides the high prevalence of sedentary behavior throughout the school day, the study also shows that children enrolled in schools with recreation room and playgrounds have reduced levels of sedentary behavior when compared to those who attend schools that do not have these facilities. In this scenario, we can suggest the development of new studies in the school environment, introducing knowledge, involving parents/guardians, and with the possibility of changing the surroundings to avoid excessive sedentary behavior. The school has a great potential for interventions related to this theme, given that educational^40^ and environmental strategies, such as the introduction height-adjustable chairs,^41^ have promising results in schoolchildren and adolescents.

### Methodological aspects

In regard to measurement instruments, considering the difficulty of preschool children in answering questionnaires,^42^ confirmed by the information that all questionnaires adopted in the summary were administered to parents, we underline that good part of the articles used accelerometers to measure physical activity and/or sedentary behavior in the populations of interest, providing a more accurate estimate of these behaviors.

However, the differences among the devices used, as well as the criteria and strategies adopted for the measurements, limits more direct comparisons among the results. This fact corroborates the need to deepen the knowledge about the tools (validation and calibration aspects) and the most appropriate criteria to evaluate physical activity and sedentary behavior objectively,^43^ so that the measurements can be more comparable, as other investigations indicate.^3^ ^,^ ^34^

In this regard, we highlight the evidence produced by the article by Lima et al., which suggests that the most appropriate criterion is monitoring for three days a week with measurements of ten hours per day, in order not to underestimate the data and avoid sample loss.^24^ In addition to objective measurements, the use of questionnaires should not be ruled out, as they can help identify the activities (for instance, which physical activities or sedentary behaviors), as well as the respective places where they happen.

### Notes for future studies

Besides the notes made in previous topics, we recommend that future researches involve larger samples, aiming at a better representation of the evidence, as one of the main weakness of the assessment for risk of bias was the lack of reports on sample representativeness. Investigations on the theme in countries other than Argentina, Brazil, and Chile are also essential to allow the development of a future summary with data from different locations and populations. Considering the data of this summary, we can also recommend the development of strategies and technologies to monitor and survey levels of physical activity and sedentary behavior in this age group in the continent.^44^

### Limitations

The main limitation of this review was not performing a preliminary survey of all South American scientific journals not indexed in the researched databases, which might have resulted in the non-evaluation of potential articles. Nevertheless, additional searches on the site Google Scholar were performed in Spanish, English, and Portuguese to avoid further effects of this limitation. We also emphasize that investigations from only three countries provided data for the summary, as well as the presence of a large number of studies that did not report their sample representativeness.

Lastly, although the summary has pointed to high exposure to sedentary behavior in preschool children, with particular focus on screen time, it is essential to broaden the discussion and approximate the methods used to assess physical activity and sedentary behavior, making the evidence more comparable and strong, so as to elaborate preventive strategies and actions
